# The immunosuppressive tumor microenvironment in glioblastoma

**DOI:** 10.3389/fimmu.2026.1806996

**Published:** 2026-07-02

**Authors:** Jing Yang, Jie Li, Ming Jin, Yongping Lu, Li Zhang

**Affiliations:** 1 Department of Emergency Medicine, Guangyuan Central Hospital, Guangyuan, Sichuan, China; 2 Scientific Research and Innovation Center, Guangyuan Central Hospital, Guangyuan, Sichuan, China; 3 Department of Cardiology, Guangyuan Central Hospital, Guangyuan, Sichuan, China

**Keywords:** glioblastoma, immunosuppression, immunotherapy, myeloid-derived suppressor cells, T-cell dysfunction, tumor microenvironment, tumor-associated macrophages

## Abstract

Glioblastoma (GBM) remains one of the most lethal primary brain tumors, with limited therapeutic improvement despite maximal surgical resection, radiotherapy, and temozolomide. A major barrier to durable treatment response is the profoundly immunosuppressive tumor microenvironment, which is characterized by immune exclusion, defective antigen presentation, myeloid dominance, and severe T-cell dysfunction. Tumor-associated macrophages, resident microglia, myeloid-derived suppressor cells, neutrophils, regulatory T cells, and glioma-derived extracellular vesicles collectively establish a suppressive niche through cytokine signaling, metabolic restriction, checkpoint ligand expression, impaired phagocytosis, and extracellular matrix remodeling. Key pathways, including TGF-β/SMAD, IL-10/STAT3, IDO–kynurenine metabolism, arginase-1-mediated amino acid depletion, adenosine signaling, hypoxia–HIF-1α activation, and VEGF-driven vascular dysfunction, converge to prevent effective antitumor immunity. This review summarizes the cellular and molecular mechanisms underlying immune suppression in GBM and discusses emerging therapeutic strategies, including myeloid reprogramming, phagocytosis checkpoint blockade, neutrophil and NET targeting, cellular immunotherapy, checkpoint blockade combinations, and metabolic intervention. Understanding these interconnected barriers may guide rational multimodal strategies to convert immune-excluded GBM into immune-responsive disease.

## 1 Introduction

Glioblastoma (GBM) is the most aggressive primary malignant tumor of the central nervous system and remains associated with dismal clinical outcomes despite maximal surgical resection followed by radiotherapy and temozolomide (1, 2). Unlike several extracranial malignancies in which immune checkpoint blockade has reshaped treatment paradigms, GBM has shown only limited and inconsistent responses to immunotherapy (3, 4). This therapeutic resistance is largely attributable to its profoundly immunosuppressive and immune-excluded tumor microenvironment, characterized by sparse cytotoxic T-cell infiltration, impaired antigen presentation, blood–brain barrier-associated trafficking limitations, and dominant myeloid-cell accumulation (5, 6). Tumor-associated macrophages, resident microglia, myeloid-derived suppressor cells, neutrophils, and regulatory T cells interact with glioma cells to establish a suppressive immune network that prevents effective antitumor immunity (7, 8).

At the molecular level, GBM immune evasion is maintained by interconnected cytokine, metabolic, vascular, and checkpoint pathways (9, 10). TGF-β/SMAD and IL-10/STAT3 signaling suppress effector lymphocyte function and reinforce tolerogenic myeloid phenotypes, whereas IDO-mediated tryptophan catabolism, arginase-1-dependent arginine depletion, and CD39/CD73-driven adenosine accumulation metabolically restrict T-cell activation (8, 11). Hypoxia-induced HIF-1α and VEGF signaling promote abnormal vasculature, macrophage polarization, and immune exclusion (12, 13). These overlapping barriers explain why single-agent immunotherapy has rarely achieved durable benefit in GBM (14). Therefore, a comprehensive understanding of the cellular and molecular architecture of GBM immunosuppression is essential for developing rational combination strategies that reprogram the tumor microenvironment and improve therapeutic responsiveness.

## 2 Immune suppressive microenvironment in the glioblastoma

### 2.1 Macrophages and microglia in glioblastoma

Tumor-associated macrophages (TAMs) and microglia, derived respectively from yolk-sac-resident microglia and bone marrow-derived monocytes, constitute the predominant immune compartment in glioblastoma (15, 16). These lineages exhibit distinct spatial distributions: monocyte-derived macrophages preferentially localize to perivascular and necrotic zones shaped by hypoxia and tissue damage, whereas resident microglia accumulate at the invasive tumor margin (17, 18). This anatomical segregation bears direct clinical relevance, as the infiltrative edge represents both the principal reservoir of residual disease post-resection and a site of pronounced immune exclusion (19, 20). Myeloid cells orchestrate adaptive immune suppression through multiple convergent mechanisms. Soluble mediators, including IL-10 and TGF-β, attenuate Th1 polarization and cytotoxic lymphocyte activity while reinforcing pro-invasive, pro-angiogenic tumor phenotypes (21). Concurrently, diminished MHC class II expression and impaired co-stimulatory signaling compromise antigen presentation, thereby limiting effective priming of tumor-specific T cells (22, 23). At the tumor–immune interface, myeloid-expressed checkpoint ligands such as PD-L1 and VISTA directly inhibit PD-1^+^ lymphocytes, sustaining local immunosuppression (24–26). They are reinforced by glioma-derived signals that promote a wound healing state, including CSF-1 and IL-34 trophic cues and chemokine circuits that recruit additional suppressive myeloid cells (27–29).

Innate immune evasion further extends to phagocytosis regulation. Glioma cells frequently upregulate CD47, which engages SIRPα on macrophages and microglia to deliver an anti-phagocytic “don’t-eat-me” signal (30, 31). Preclinical studies demonstrate that disruption of CD47–SIRPα signaling enhances tumor cell engulfment and correlates with prolonged survival in experimental models, implicating defective innate clearance in glioma immune escape (32). Beyond direct cytoreduction, restored phagocytosis may augment antigen availability for cross-presentation, thereby potentiating adaptive priming—particularly when combined with interventions that support dendritic cell function and T-cell expansion (30, 33). Tumor genetics further modulates myeloid recruitment and functional polarization. IDH-mutant gliomas typically exhibit reduced immune infiltration and lower checkpoint ligand expression relative to IDH-wildtype GBM, reflecting divergent immune-regulatory landscapes (20, 34, 35). The oncometabolite 2-hydroxyglutarate has been associated with altered myeloid trafficking and a less inflammatory microglial phenotype, contributing to sustained immunosuppression and diminished responsiveness to immunotherapy (36). Conversely, IDH-wildtype GBM commonly displays robust macrophage recruitment coupled with inflammatory signaling that is co-opted toward immunosuppressive outputs—including IL-10/TGF-β secretion, impaired antigen presentation, and checkpoint-mediated inhibition (37, 38). These molecular distinctions underscore that glioma immunotherapy cannot presuppose a uniform microenvironment; rather, effective strategies must be tailored to subtype-specific immune architectures.

### 2.2 Neutrophils and extracellular traps in immune exclusion

Within the myeloid-dominated glioma microenvironment, neutrophils function not as isolated innate effectors but as downstream amplifiers of immune exclusion, reinforcing suppression initiated by macrophages and microglia (39–41). Their accumulation correlates with aggressive disease and therapeutic resistance, particularly in recurrent GBM. Clinically, elevated neutrophil-to-lymphocyte ratios serve as a prognostic indicator of poor survival, reflecting systemic myelopoietic skewing and concurrent lymphocyte deficiency (42, 43). Neutrophil recruitment is primarily governed by tumor- and myeloid-derived chemokines, notably CXCL2 and CXCL8, which signal through CXCR2 on circulating neutrophils (44, 45). Single-cell transcriptomic analyses reveal that therapy-resistant states can intensify these chemokine program, thereby augmenting neutrophil influx and consolidating a suppressive niche during disease recurrence (46). Once infiltrated, neutrophils impair T-cell function via metabolic and oxidative mechanisms: arginase-1 depletes extracellular arginine essential for T-cell proliferation, while reactive oxygen species disrupt T-cell receptor signaling and cytotoxic capacity, even in the presence of antigen recognition (40, 47, 48). Beyond direct immunosuppression, neutrophils contribute to structural remodeling that perpetuates immune exclusion. They secrete matrix-degrading enzymes and pro-angiogenic factors that facilitate tumor invasion and alter vascular architecture, thereby limiting lymphocyte access to tumor nests. Neutrophil extracellular traps (NETs), composed of decondensed chromatin scaffolds adorned with proteases, histones, and myeloperoxidase, can persist post-resection and create permissive conduits for residual tumor cell migration (49–51). This NET-mediated matrix remodeling offers a mechanistic explanation for rapid local regrowth despite aggressive cytoreduction, positioning neutrophils as key orchestrators of both functional and physical barriers to effective anti-tumor immunity.

### 2.3 T cell dysfunction in glioblastoma

T-cell dysfunction in GBM represents the terminal adaptive consequence of upstream myeloid dominance, neutrophil-mediated tissue remodeling, and metabolic checkpoint activation. Most GBMs harbor sparse tumor-infiltrating lymphocytes, and those that penetrate the tumor frequently exhibit dysfunctional phenotypes characterized by impaired cytotoxicity and proliferative capacity (25, 52, 53). Intratumoral T cells commonly co-express PD-1 with multiple inhibitory receptors—including TIM-3, LAG-3, TIGIT, and CTLA-4—reflecting chronic antigen exposure under sustained immunosuppression (54, 55). Such co-expression correlates with diminished cytokine production, reduced clonal expansion, and compromised tumor-cell killing, defining a transcriptionally exhausted state that limits endogenous immune control. Spatial architecture further constrains T-cell efficacy (56). Myeloid-enriched perivascular niches can sequester lymphocytes and impede their infiltration into tumor-cell-dense regions, generating an immune-excluded topology even when T cells are detectable (57, 58). This compartmentalization may confound biomarker interpretation, as the mere presence of immune cells does not guarantee productive tumor engagement.

Regulatory T cells (Tregs) reinforce this suppressive landscape and bridge adaptive dysfunction to chemokine-driven myeloid recruitment. Gliomas secrete CCL2, CCL22, and CXCL12, which coordinate the trafficking of Tregs alongside suppressive myeloid subsets (59, 60). CCL2 is particularly consequential, attracting both Tregs and CCR2^+^ monocytic suppressor cells; elevated CCL2 signaling independently correlates with poorer clinical outcomes. Within the tumor, Tregs suppress effector responses via IL-10 and TGF-β secretion and through CTLA-4-mediated disruption of antigen-presenting cell co-stimulation (60–63). These functions are amplified by metabolic constraints: hypoxia and adenosine accumulation favor Treg differentiation while impairing the fitness of infiltrating effector cells. Central nervous system-specific barriers further compound these challenges. Even with partial blood–brain barrier disruption, lymphocyte entry remains limited in infiltrative regions, and inefficient antigen drainage to cervical lymph nodes may curtail priming efficiency (64–66). Standard chemoradiotherapy frequently induces prolonged lymphopenia, depleting the effector pool available for subsequent immunotherapeutic interventions (67, 68). Concomitant high-dose corticosteroid use—often required for oedema management—further blunts T-cell activation and may attenuate the efficacy of PD-1 pathway inhibition, particularly when administered near checkpoint blockade initiation (69, 70). These features explain why immunotherapies reliant on endogenous priming often underperform in GBM unless integrated with strategies that enhance local antigen exposure, mitigate immunosuppressive cues, and preserve effector-cell fitness.

### 2.4 MDSCs and soluble mediators in glioblastoma

Myeloid-derived suppressor cells (MDSCs) consolidate this immunosuppressive hierarchy by translating inflammatory recruitment into sustained biochemical and cellular restraint (54, 61). Expanding systemically and infiltrating the tumor as polymorphonuclear and monocytic subsets, MDSCs impair T-cell receptor signaling, survival, and trafficking via arginase-1, nitric oxide, and reactive oxygen species (40, 71). Beyond metabolic disruption, they directly engage inhibitory pathways on exhausted lymphocytes through surface expression of PD-L1 and CD155 (72). MDSCs further amplify suppression through reciprocal crosstalk with Tregs and by differentiating into macrophage-like populations that perpetuate cytokine-mediated inhibition and blunt antigen presentation (61). Functionally, they reinforce immune exclusion by secreting neutrophil-attracting chemokines and promoting aberrant angiogenesis, thereby physically limiting effector infiltration (73). This systemic expansion also underlies the peripheral immune dysfunction characteristic of GBM, manifesting as profound lymphopenia and blunted T-cell responsiveness to stimulation.

**Figure 1:**
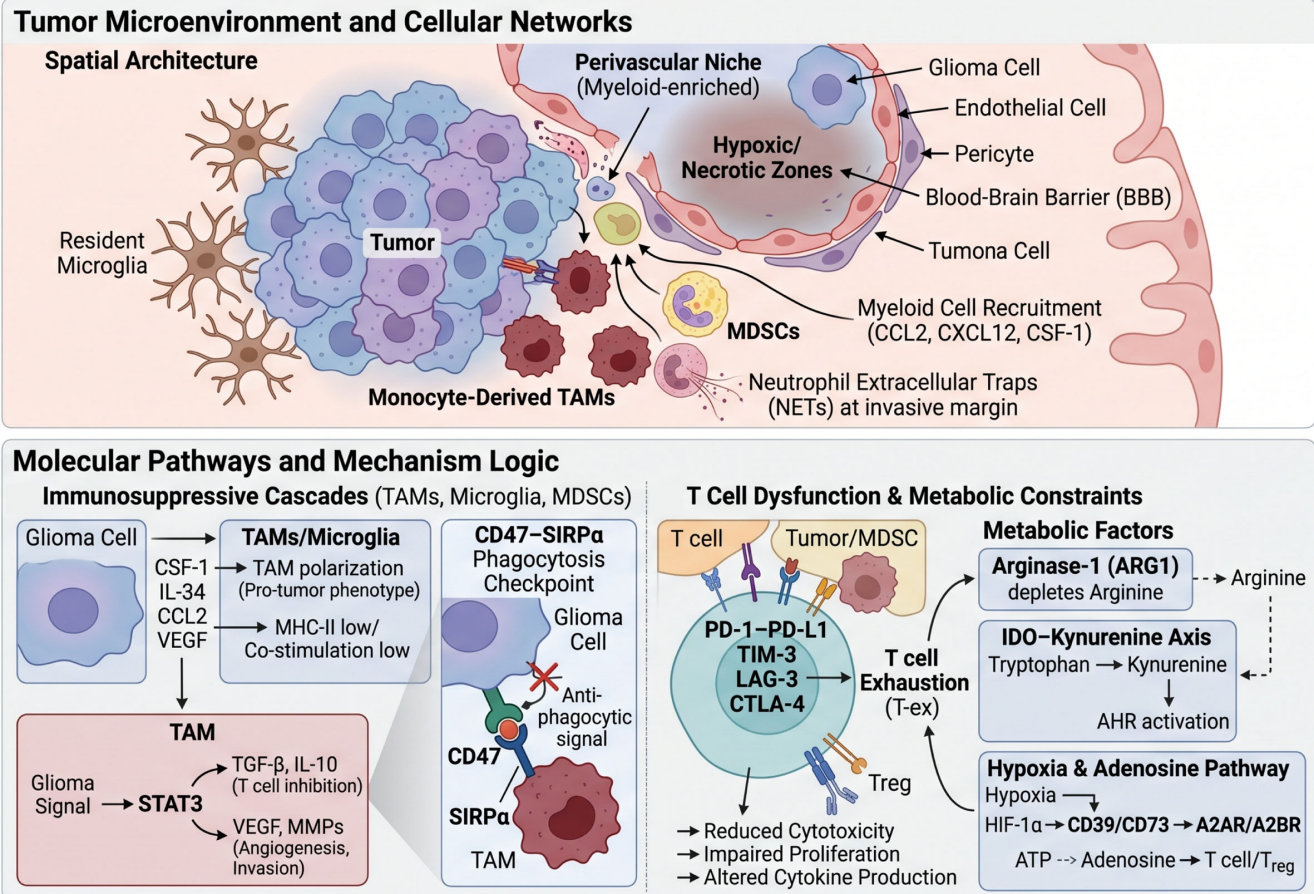
Immunosuppressive barriers and therapeutic strategies in glioblastoma.

Soluble cytokines and metabolic constraints impose additional barriers on any effector cell that penetrates the tumor niche. TGF-β suppresses cytotoxic T-cell and natural killer-cell activity while promoting invasive and stem-like phenotypes, directly coupling immune evasion to tumor aggressiveness (74, 75). Concurrently, IL-10 stabilizes tolerogenic myeloid states and diminishes antigen-presenting capacity, reinforcing a permissive microenvironment. Metabolic checkpoint activation further compounds these effects: IDO-mediated tryptophan catabolism depletes essential nutrients and generates kynurenine metabolites that drive T-cell skewing toward regulatory states while suppressing dendritic cell function (76, 77). Hypoxia intensifies this landscape by activating HIF-1α-driven transcriptional programs that favor angiogenesis and inhibit effector differentiation. Compounded by lactate accumulation, glucose restriction, and VEGF-mediated vascular dysfunction, the resulting metabolic stress and aberrant vasculature transform immune exclusion into a self-sustaining state, effectively neutralizing anti-tumor responses even in antigen-rich regions (78, 79). Extracellular vesicles (EVs) extend these suppressive mechanisms beyond direct cell–cell contact, enabling long-range communication within and outside the central nervous system. Glioma-derived EVs traverse the blood–brain barrier and are readily detectable in peripheral blood and cerebrospinal fluid, positioning them as valuable biomarkers of tumor immune status (80, 81). EVs transport immunomodulatory cargo that systemically reinforces tolerance: exosomal PD-L1 engages PD-1^+^ T cells at a distance, with circulating levels rising post-radiotherapy to mediate adaptive resistance and treatment-induced immune escape (82, 83). Additionally, EV-associated microRNAs (miR-21, miR-222) reprogram microglia and myeloid cells toward anti-inflammatory, IL-10/IL-6-dominant phenotypes, while oncogenic protein payloads activate STAT3-dependent suppressive program (84, 85) (**Figure 1**).

## 3 Potential therapeutic approaches to reprogram glioma immunity

### 3.1 Myeloid reprogramming and phagocytosis restoration

Given the central role of macrophages and microglia in orchestrating immunosuppression, therapeutic strategies targeting myeloid reprogramming have emerged as a rational approach in GBM. Inhibition of the CSF-1 receptor (CSF-1R) aims to deplete or repolarize these populations, and preclinical studies demonstrate that CSF-1R blockade attenuates tumor-supportive programs and slows disease progression, validating myeloid plasticity as a therapeutic target (86, 87). However, clinical activity in recurrent GBM has remained modest despite confirmed target engagement, suggesting compensatory recruitment via redundant trophic signals and persistence of parallel suppressive pathways (88). These findings underscore the necessity of combination strategies over monotherapeutic depletion, as isolated CSF-1R inhibition may be circumvented by alternative chemokine axes and sustained monocyte influx. Targeting recruitment pathways such as CCL2–CCR2 offers a conceptually complementary approach, potentially reducing suppressive macrophages and monocytic MDSCs while concurrently limiting regulatory T-cell trafficking, thereby reshaping the immune landscape prior to cytotoxic intervention (59, 60). Phagocytosis checkpoint blockade represents a parallel strategy to restore innate anti-tumor activity. Disruption of the CD47–SIRPα interaction reinvigorates macrophage- and microglia-mediated engulfment of glioma cells and enhances antigen processing (30, 38). Early clinical experience in pediatric brain tumors supports the feasibility of CD47-targeted therapy, though on-target anemia remains an expected toxicity given constitutive CD47 expression on erythrocytes (89, 90). The therapeutic potential of phagocytosis checkpoint inhibition may be maximized when integrated with modalities that promote T-cell priming or persistence—a hypothesis under active investigation. Accordingly, this approach aligns synergistically with strategies that provide local antigen exposure, attenuate IL-10/TGF-β signaling, or remodel adenosine-rich hypoxic niches, collectively fostering a microenvironment permissive to adaptive immune engagement (91, 92).

### 3.2 Neutrophil and NET-directed strategies

Targeting neutrophil recruitment and extracellular trap formation offers a complementary strategy to dismantle both functional and physical barriers within the GBM microenvironment. Inhibition of the CXCR2 chemokine receptor effectively curtails neutrophil infiltration in preclinical GBM models, concurrently augmenting intratumoral CD8^+^ T-cell densities and extending survival (39, 93). CXCR2 blockade attenuates CXCL1/CXCL2-driven granulocytic influx, mitigates arginase-1- and ROS-mediated T-cell suppression, and disrupts the formation of neutrophil-enriched perivascular and necrotic niches that physically impede lymphocyte penetration (94). Given the marked functional plasticity of neutrophils, therapeutic intervention should prioritize the selective suppression of pro-tumorigenic granulocytic programs over wholesale depletion, thereby preserving essential antimicrobial host defense (39, 95). Neutrophil extracellular traps (NETs) represent as another potential target within this axis. Composed of decondensed chromatin scaffolds adorned with proteases, histones, myeloperoxidase, and neutrophil elastase, NETs remodel the extracellular matrix, facilitate tumor cell adhesion and migration, and perpetuate sterile inflammation following surgical resection or radiotherapy (43, 96). Preclinical studies demonstrate that DNase-mediated NET degradation attenuates local invasion and reduces post-surgical recurrence. Upstream inhibition of peptidylarginine deiminase 4 (PAD4), the enzyme catalyzing histone citrullination essential for NETosis, further suppresses NET formation, thereby mitigating matrix remodeling, vascular dysfunction, and immune exclusion (43, 97). Clinically, these interventions may yield maximal efficacy when deployed during perioperative or early post-radiotherapy windows, periods characterized by acute tissue injury, hypoxia, and chemokine surges that collectively amplify neutrophil recruitment and NET deposition.

### 3.3 Cellular immunotherapy and barriers to durability

Adoptive cell therapy seeks to circumvent defective endogenous priming by infusing tumor-reactive effectors directly into the central nervous system. Locoregional delivery of IL13Rα2-directed CAR T cells has demonstrated profound clinical responses in recurrent GBM, establishing proof-of-concept for this modality (98, 99). Similarly, EGFRvIII-targeted CAR T cells successfully trafficked to tumor sites, though clinical efficacy was ultimately constrained by antigen loss and rapid adaptive remodeling of the microenvironment, including upregulation of inhibitory ligands and intensified myeloid suppression (100, 101). These outcomes highlight antigen heterogeneity and immune escape as fundamental barriers in GBM. Mosaic antigen expression enables antigen-negative clones to evade selective pressure, while infiltring cellular products rapidly encounter a metabolically hostile and immunosuppressive niche (102, 103). Suppressive myeloid populations, regulatory T cells, hypoxia, lactate accumulation, adenosine signaling, and nutrient deprivation collectively impair CAR T-cell proliferation, cytokine secretion, cytotoxic granule release, and memory formation (104, 105). Persistent antigen exposure further drives transcriptional exhaustion program characterized by sustained PD-1, TIM-3, LAG-3, and TOX expression, ultimately compromising effector fitness and limiting long-term tumor control (106).

To overcome these limitations, next-generation platforms are increasingly incorporating multi-antigen targeting, logic-gated receptor architectures, and constitutive secretion of immunomodulatory cytokines or checkpoint-blocking molecules (107, 108). Dual- and tri-specific CAR designs, alongside engineered resistance to metabolic stress, aim to prevent early functional collapse and enhance persistence within the glioma niche (101, 108). Natural killer cell-based therapies offer a complementary strategy less dependent on classical MHC-restricted antigen presentation, though their cytotoxic function and survival remain similarly constrained by the suppressive microenvironment (109, 110). Across cellular modalities, locoregional administration, including intratumoral or intraventricular delivery, is increasingly employed to bypass blood–brain barrier trafficking limitations and achieve therapeutic concentrations within CNS compartments most relevant to disease recurrence.

### 3.4 Checkpoint blockade, priming platforms, and metabolic combinations

Checkpoint inhibitor monotherapy has failed to improve survival in GBM, an outcome consistent with low baseline T-cell infiltration, defective antigen presentation, and dominant myeloid-mediated suppression (111). Nevertheless, therapeutic timing and contextual priming remain critical determinants of efficacy. Neoadjuvant PD-1 blockade, for instance, has been associated with enhanced intratumoral immune activation signatures, supporting the rational evaluation of perioperative windows and combinations that augment antigen exposure and dendritic cell priming (112, 113). Local interventions offer promising avenues to convert immune- exclusion tumors into immunoresponsive lesions. Focal radiotherapy can increase neoantigen release and upregulate chemokines that recruit lymphocytes, while intratumoral delivery of cytokines or pattern-recognition receptor agonists can drive dendritic cell maturation and type I interferon programs (114, 115). However, standard chemoradiotherapy frequently induces prolonged lymphopenia, and concomitant corticosteroid use—often required for oedema management—further depletes the effector pool upon which checkpoint blockade depends (67, 116). Strategies that minimize steroid exposure, preserve lymphocyte fitness, and concurrently reprogram myeloid compartments may therefore enhance therapeutic efficacy, though their relative contributions warrant systematic definition (69, 117). Notably, integrating priming interventions with vascular normalization and myeloid targeting may improve lymphocyte penetration into infiltrative margins, the principal source of disease recurrence.

Metabolic checkpoint inhibition is increasingly incorporated into multimodal designs to counteract the immunosuppressive biochemistry of the GBM niche. Blockade of the adenosine pathway—via A_2_A receptor antagonism or CD39/CD73 inhibition—can restore effector T-cell function within hypoxic regions and synergize with PD-1 blockade in preclinical models (118, 119). Similarly, targeting the IDO–kynurenine axis or TGF-β signaling aims to alleviate metabolic and cytokine-driven suppression, though durable clinical benefit likely requires pairing with modalities that enhance effector generation or tumor trafficking (120, 121). Current evidence suggests that checkpoint blockade may achieve maximal efficacy when embedded within multimodal regimens that concurrently increase antigen availability, improve immune-cell access to tumor compartments, and sustain T-cell function amidst the metabolically restrictive glioma microenvironment (122, 123). Validation of this integrative framework remains a priority for prospective clinical investigation.

## 4 Conclusion

The immunosuppressive tumor microenvironment is a central determinant of glioblastoma progression, therapeutic resistance, and failure of immunotherapy. Unlike immunologically “hot” tumors, GBM is shaped by profound immune exclusion, limited effector T-cell infiltration, impaired antigen presentation, and dominant myeloid-mediated suppression. Tumor-associated macrophages, microglia, MDSCs, neutrophils, and Tregs form an interconnected suppressive network that restrains cytotoxic immunity through inhibitory cytokines, checkpoint ligands, metabolic deprivation, abnormal vasculature, and extracellular matrix remodeling. In parallel, glioma-derived extracellular vesicles and hypoxia-driven signaling extend immune suppression beyond the local tumor niche and contribute to systemic immune dysfunction. These features explain why single-agent checkpoint blockade has shown limited efficacy and highlight the need to move beyond approaches that target only one immune pathway.

Future therapeutic development should prioritize rational combination strategies that simultaneously enhance antigen presentation, restore innate and adaptive immune function, and dismantle physical and metabolic barriers to immune infiltration. Myeloid reprogramming, CD47–SIRPα blockade, CXCR2 or NET-directed intervention, adenosine and IDO pathway inhibition, vascular normalization, and locally delivered cellular immunotherapies represent promising directions. However, durable benefit will likely require precise patient stratification based on tumor genetics, spatial immune architecture, treatment history, corticosteroid exposure, and systemic immune status. Integrating single-cell sequencing, spatial transcriptomics, proteomics, and longitudinal immune monitoring may enable a more refined understanding of GBM immune heterogeneity and identify actionable vulnerabilities. Ultimately, effective immunotherapy for GBM will depend not on isolated immune activation, but on coordinated remodeling of the tumor ecosystem to convert immune-excluded lesions into immunologically accessible and therapeutically responsive tumors.
